# Enteral Linezolid as an Effective Option to Treat an Extremely Preterm Infant with *Bacillus cereus* Sepsis

**DOI:** 10.3390/children9030415

**Published:** 2022-03-15

**Authors:** Chiara Minotti, Luca Bonadies, Cecilia Liberati, Marica De Pieri, Carlo Giaquinto, Eugenio Baraldi, Daniele Donà

**Affiliations:** 1 Division of Pediatric Infectious Diseases, Department of Women’s and Children’s Health, University of Padova, 35128 Padova, Italy; liberaticecilia@gmail.com (C.L.); marica.depieri@aopd.veneto.it (M.D.P.); carlo.giaquinto@unipd.it (C.G.); daniele.dona@unipd.it (D.D.); 2 Neonatal Intensive Care Unit, Department of Women’s and Children’s Health, University of Padova, 35128 Padova, Italy; lucabonadies@hotmail.it (L.B.); eugenio.baraldi@unipd.it (E.B.)

**Keywords:** preterm, newborn, linezolid, *B. cereus*, invasive infection, sepsis

## Abstract

We report the safe and effective use of oral linezolid for treatment of *Bacillus cereus* sepsis in an extremely preterm neonate, previously fed with human donor milk, in which a *Brevibacillus* sp. was eventually found. Due to several predisposing factors, premature, very low birth weight newborns are extremely vulnerable to invasive infections by environmental pathogens. After vancomycin microbiologic treatment failure (despite adequate blood concentrations and clinical response), linezolid was chosen for its optimal enteral absorption and bioavailability, also after exhaustion of peripheral venous heritage. No adverse events were recorded, with clinical cure. We reviewed the literature on *B. cereus* infections in newborns, together with the available evidence on the use of linezolid in similar contexts.

## 1. Introduction

*Bacillus cereus* is a relatively uncommon but potentially serious pathogen that may be responsible for invasive infections in preterm newborns. It is a Gram-positive or Gram-variable, aerobic or facultatively anaerobic, spore-producing ubiquitous bacterium that can be found in the environment, including soil, dust and water sources [1]. As a human pathogen, it usually causes self-limiting food-borne illness, as well as opportunistic infections in immunocompromised hosts. Preterm newborns requiring intensive care are especially susceptible to invasive disease by environmental pathogens. However, the reported cases of invasive infections by *B. cereus* in this special population are few [1]. Linezolid is used to treat severe infections by Gram-positive bacteria, including methicillin-resistant *Staphylococcus aureus* (MRSA), as an alternative to vancomycin, but evidence for its use in the neonatal population, especially in extremely preterm infants, is scarce [2]. We report the safe and effective use of enteral linezolid for treating *B. cereus* sepsis in an extremely preterm neonate, previously fed with human donor milk, in which a *Brevibacillus* sp. was found.

## 2. Case Report

An extremely preterm male infant born at 24 weeks and one day of gestation by vaginal delivery after premature labor in suspected chorioamnionitis (pPROM longer than 48 h, maternal fever, elevated maternal C-reactive protein (CRP), unusual smell of the amniotic fluid) was admitted to the Neonatal Intensive Care Unit (NICU). His birth weight was 580 g. Apgar index was 5, 7 and 8 at 1, 5 and 10 min of life, respectively. The only anomaly at clinical examination was the presence of hypospadias.

He was intubated in the delivery room because of severe respiratory distress on non-invasive support and received intratracheal administration of surfactant a few minutes later in the NICU with consequent optimal clinical response. He then received another surfactant administration on the second day of life (DOL). The neonate failed extubation twice, on the first and seventh DOL, despite treatment with caffeine. He was pharmacologically treated (with ibuprofen) for a hemodynamically significant patent ductus arteriosus. He also underwent standard antibiotic treatment with ampicillin and netilmicin since birth for 48 h for a suspected non-microbiologically confirmed early-onset sepsis (EOS) consequent to chorioamnionitis. An umbilical venous catheter was positioned at birth and replaced with an epicutaneous-caval catheter on the sixth DOL to allow parenteral nutrition because of feeding intolerance.

He was successfully extubated and supported with non-invasive ventilation in stable clinical conditions on the sixteenth DOL. However, on the twentieth DOL, the neonate showed an abrupt clinical decay, with an increased number of apneas, marbled skin, increased oxygen needs and hypotension. In addition, the chest X-ray showed a marked reduction in the transparency of both lungs. 

The neonate was consequently intubated, a saline bolus was administered and blood chemistry tests were performed (complete blood count (CBC), CRP, blood gas analysis, blood culture). The blood gas analysis showed a mixed acidosis and hyperglycemia, CRP was slightly elevated (14.6 mg/L) and slight thrombocytopenia (122 × 10^9^/L) and anemization were discovered. Urine and bronchial aspiration were collected for culture, while lumbar puncture was postponed for clinical instability. Antibiotic therapy was promptly started with vancomycin and gentamicin as per local protocol; packed red blood cells were transfused.

The newborn showed a transitory hemodynamic and metabolic/glycemic stabilization but a progressive respiratory worsening with the need of rescue high-frequency oscillatory ventilation.

Two days later (22nd DOL), blood chemical tests were re-performed, including a second blood culture for the persistence of clinical and hemodynamic instability (hypotension), showing a further increase of CRP (44 mg/L), platelet count reduction (79 × 10^9^/L) and a new anemization. A continuous dopamine infusion was started and another transfusion with packed red blood cells was administered, with subsequent peripheral perfusion improvement. The antibiotic treatment was modified to piperacillin–tazobactam and vancomycin, removing gentamicin for the persistence of clinical symptoms and acute kidney injury signs in blood chemicals.

The infant then showed progressive clinical improvement with concurrent CRP reduction and negativization on the 30th DOL, despite the persistence of thrombocytopenia (30 × 10^9^/L) from the 21st DOL, for which he was transfused three times in one week. Dopamine support was ceased on the 21st DOL.

However, the second blood culture showed positive for a Gram-positive *Bacillus* susceptible to vancomycin, identified as a *B. cereus* (susceptible to vancomycin, MIC = 1, and linezolid, MIC = 2), that was also recovered on subsequent blood cultures of DOL 27, 29, 31, 37, 42 and 45. At that time, the patient was fed with human donor milk. Bronchial aspirate and urine culture were repeatedly negative. CRP persisted negative. Cerebrospinal fluid was collected and cultured on DOL 45 with no growth.

The epicutaneous-caval catheter was removed on DOL 25 because of the first positive blood culture. A new echo-guided centrally inserted venous catheter (CICC) was placed, after a prolonged washout, on the 33rd DOL because of the paucity of available peripheral veins, long used for antibiotic therapy and parenteral nutrition. The CICC was then removed on DOL 40 after the positive result of the blood culture of the 37th DOL. The treatment with piperacillin–tazobactam was stopped on DOL 32 after consultation with the pediatric infectious diseases specialist. Vancomycin blood levels were persistently adequate, and therefore treatment was administered up to the 45th DOL when the peripheral venous heritage was no longer usable.

At that point, with persistently positive blood cultures and without any further option for peripheral venous access, the choice fell on enteral treatment, the patient having reached full enteral feeding in the meantime. Oral linezolid via orogastric tube at a dose of 10 mg/kg/dose every eight hours was started on DOL 45 because of its optimal enteral absorption. At the same time, a progressive transition to formula milk was started. 

After this therapeutic change (on the 48th DOL), the first blood culture showed no growth for *B. cereus*. At that time, the patient was still fed with 25% of donor milk and 75% of formula milk. The blood cultures of the 52nd and 57th DOL persisted negative.

To further evaluate the persistently positive cultures for *B. cereus*, with it being sporogenous and having been found in literature reports of late-onset sepsis by *B. cereus* potentially acquired from pasteurized milk (even without confirmation), the donor milk that the infant received during the whole period was analyzed upon our request, and the growth of a *Brevibacillus* sp., belonging to the genus *Bacillus*, was found. 

The treatment with linezolid was continued for two weeks since the first negative blood culture, up to the 64th DOL. Complete blood count and kidney and liver function tests were repeatedly checked over the treatment period, without any sign of toxicity, and also during follow-up. The baby was transferred to a peripheral hospital facility after 67 days of hospitalization, still on high-flow nasal cannula respiratory support, on bottle-feeding with preterm special formula. He was discharged home one month later, still on oxygen therapy. He was diagnosed with moderate bronchopulmonary dysplasia (BPD), retinopathy of prematurity (ROP) stage 2–3 that required intravitreal treatment with anti-vascular endothelial growth factor (anti-VEGF). The baby never presented intraventricular hemorrhage; visual tests showed an improvement and auditory tests were normal at follow-up, with no evidence of organ damage (normal liver tests and creatinine values). At one-month neonatological follow-up, he still needs home oxygen therapy, with brief moments of acceptable saturation values in room air. He is otherwise thriving and does not present any particular concern from a neurological point of view. However, it is still early for the administration of specific neurodevelopmental tests. 

## 3. Discussion

To our knowledge, this is the first report of the safe and successful use of oral linezolid in an extremely preterm infant for treatment of an invasive infection by *B. cereus*. It proved to be an extremely valuable option, considering a lack of microbiological response to vancomycin and the peripheral venous heritage exhaustion. It may, therefore, be considered in cases of vancomycin failure or in the critical context of the impossibility of placement of a central venous line. Linezolid was administered as a liquid suspension by nasogastric tube, without any difficulty. The administration was well tolerated, without any modification in gastric residuals or in consistency, color or frequency of stools and no vomiting, which are all possible side-effects. The patient was strictly monitored for modifications of abdominal examination, in the awareness that the local action of linezolid could affect the enteral microbiota and somehow increase the risk of necrotizing enterocolitis, reducing the presence of *Streptococcus* spp. and *Staphylococcus* spp. [3]. Linezolid is described as related to allergic reactions and possible liver damage with elevated transaminases. We therefore routinely monitored these factors and did not find any significant modification.

Linezolid is an oxazolidinone antibiotic inhibiting bacterial protein synthesis. Its mechanism of action is specific to this class, and there is no cross-resistance with the other antibiotics inhibiting protein synthesis. It is active against Gram-positive bacteria, including *Streptococcus* spp., MRSA and vancomycin-resistant *Enterococcus* (VRE). Its oral absorption and bioavailability are outstanding (100%, slightly reduced by the presence of food), and serum peak concentrations are reached within one or two hours [4,5]. Its half-life is 5.6 h in preterm patients, 3 h in full-term newborns during the first week of life and 1.5 h in older infants. It binds 31% to plasmatic proteins and is mostly eliminated extra-renally (65%), avoiding renal side effects, more common with vancomycin [2]. Linezolid is usually indicated as a valuable alternative to vancomycin in case of severe infections by MRSA. Most of the data regarding pharmacokinetics, efficacy and tolerability derive from studies on adults, and few data on the pediatric and especially neonatal population are available. As reported by Jungbluth, the pharmacokinetics of linezolid varies with age, as patients younger than 12 years have a smaller area under the drug concentration–time curve, faster clearance and a shorter elimination half-life than adults but with closer clearance rates between neonates and adults [2]. The recommended dose for the neonatal population is 10 mg/kg/dose for either oral or intravenous administration, to be administered every 12 or 8 h according to weight and post-natal age (intervals of 12 h are recommended for gestational age < 34 weeks), following one of the most widely used infectious diseases handbooks and data from the literature [6,7,8]. These findings are supported by studies on linezolid pharmacokinetics in neonates, stratified according to gestational age and post-natal age [6,9,10], demonstrating, at age stratification, lower clearance values for neonates aged less than eight days, and thus justifying the adoption of longer intervals (two daily administrations). The available data from the pediatric and neonatal population show a favorable tolerability, safety and efficacy profile [11,12]. Thibault reported the good efficacy, safety and tolerability of intravenous linezolid in a retrospective cohort of premature infants at standard doses [13]. All infants achieved the target area under the concentration–time curve/minimum inhibitory concentration, and the main factor influencing clearance was post-natal age. Chiappini and colleagues led a systematic review on the use of linezolid in the pediatric population, reporting its safety and tolerability for treating skin infections, bacteremia and pneumonia, with elevated clinical cure rates up to 93% [14]. DeVille and colleagues described a cohort of 63 neonates with hospital-acquired pneumonia, complicated skin infections, bacteremia or invasive infections by resistant Gram-positive bacteria after randomization 1:2 between intravenous vancomycin vs. linezolid [15]. Treatment duration ranged from 10 to 28 days, with higher cure rates for linezolid, even though not statistically significant. There were no significant differences regarding adverse events. Linezolid was overall well-tolerated, with a good efficacy profile in this population. Sicard reported satisfactory plasma concentrations after linezolid intravenous and oral administration concerning drug MICs in extremely preterm infants [16]. The authors also reported cases of myelosuppression in this vulnerable population, with resolution after treatment discontinuation. The most important reported adverse effect for prolonged treatments, lasting more than 10–14 days, is myelosuppression, with thrombocytopenia, anemia, leucopenia or pancytopenia. Some authors also described a relation between thrombocytopenia and higher minimum drug concentrations, suggesting that serum concentrations may also be involved in causing thrombocytopenia [17,18,19]. Other reported adverse events include neurologic complications (peripheral neuropathies, optical neuritis, seizures), fever, dyspnea, hypertension, hypercreatininemia and cutaneous manifestations, such as rash. Gastrointestinal effects are also described, such as vomiting/diarrhea, abdominal tenderness, hypertransaminasemia, pancreatitis and pseudomembranous colitis [4]. Last, a recent study by Li and colleagues described linezolid population pharmacokinetics with a one-compartment model with first order elimination along with body weight and eGFR as significant covariates for dosing optimization, starting from the data of 112 pediatric patients, including neonates [18]. For children older than two years, they suggested a higher dose of 15 to 20 mg/kg every 8 h for infections by pathogens with MIC ≥ 2 mg/L to reach the pharmacokinetics/pharmacodynamics target. However, we did not find evidence suggesting different dosing for the neonatal population, other than the regimen we adopted for our patient. Linezolid was indeed a valuable option for its optimal oral bioavailability and the scarce options for IV accesses. Overall, the treatment was effective in infection eradication, safe and tolerable, with no adverse effects after two weeks. No evidence was found in the literature describing linezolid for treating invasive infections by *B. cereus* in neonates. Hilliard described a case of *B. cereus* bacteremia in an extremely preterm newborn that was successfully treated with a combination of vancomycin, tobramycin, clindamycin and meropenem for ten days [1]. The survival of preterm, very low birth weight newborns has increased over the years due to advances in neonatal intensive care. This vulnerable population remains extremely susceptible to invasive infections, even by environmental pathogens, because of several predisposing factors, such as prolonged mechanical ventilation, parenteral nutrition and long-term intravascular accesses. However, the reported cases of *B. cereus* infections in infants remain rare, with frequent cases of culture positivities attributed to contamination. Not being familiar with *B. cereus* as a causative late-onset sepsis pathogen may lead to some degree of scepticism. Other *Bacillus* species are thought to be more common, and there is also a belief in incomplete attempts of microbiological identification of *Bacillus* species [1]. Published *B. cereus* NICU outbreak reports identified the environmental presence of the pathogen (on the hands of nursing staff, mechanical ventilation equipment, central catheter lines, feeding tubes) [1,20,21,22]. In our case, there were several subsequent findings of *B. cereus* growth from blood cultures and the identification of *Brevibacillus* sp. from donor milk samples. Human donor milk was administered since birth (as a minimal enteral feeding) and when the daily volume was adequately tolerated, it was progressively increased (by 10–20 mL/kg/day), reaching full enteral feeding (160 mL/kg/die) on the 45th DOL, when it was slowly shifted to formula because of concerns of possible contamination. The use of human donor milk is supported in extremely preterm infants by important evidence of improved outcomes compared with formula milk [23]. The microbiological analysis of donor milk was performed afterwards upon our request. In our unit, donor breast milk is routinely managed following the recommendations from the European Milk Bank Association [24] with the Holder Pasteurization (HoP) technique. It is collected by donor mothers, then frozen at −20 °C maximum 24 h after collection. It is delivered to the local milk bank, where it is stored frozen for a maximum of 3 months. When needed, the milk is slowly unfrozen, pasteurized (62.5 °C for 30 min) in sterile bottles and stored in a fridge. Right before the administration, it is warmed and administered by enteral feeding syringes a maximum of 24 h after pasteurization. The process of EMB management has not been changed after this episode.

Cases of suspected involvement of human donor milk are reported, with hardly any confirmation. Lewin et al. described two cases of *B. cereus* neonatal infection, trying to determine the potential causal role of donor milk as a source of infection, but without a certain correspondence [25]. In the first case that was unsuccessfully treated with linezolid, meropenem and vancomycin, with eventual death due to sepsis complications, two genetically different *B. cereus* species were identified in the patient’s blood cultures and the milk samples. In the second case, a *Brevibacillus* sp. was isolated from banked milk, similarly to our case. The taxonomic relationship between *Bacillus* species is partially unclear. In laboratory diagnostics, differentiation among species may be difficult, with many phenotypic tests used for distinction, although sometimes only a single feature differentiates species. The genus *Bacillus* consists of three wide groups, according to spore and sporangium morphology [26]. *B. cereus* group (group 1) includes eight species and produces two kinds of toxins; one is thermostable and emetic, the other is thermolabile and can cause diarrhea. Moreover, *B. cereus* can shift to the vegetative form to spore in case of unfavorable environmental conditions, which can ensure prolonged survival [27]. *Brevibacillus brevis* belongs to group 2 species (Gram-variable, swollen sporangia). 

It is important to underline that human milk is of paramount importance for preterm newborns’ nutrition, with several advantages for short- and long-term outcomes, especially in terms of illness and infection development. Human donor milk remains the second-best option after fresh human milk. However, to date, there is no consensus on the prevention and management of contamination and microbiological analysis before and after donor human milk pasteurization, with a risk of becoming a potential source of infection for vulnerable infants. As pointed out in a recent study, routine microbiological analysis of a single donor pool after the pasteurization process could help avoid *B. cereus* contamination [28]. 
